# Association between nursing work environment, burnout, and turnover intention: A cross-sectional study in Greece

**DOI:** 10.3934/publichealth.2025054

**Published:** 2025-11-13

**Authors:** Ioannis Moisoglou, Aglaia Katsiroumpa, Evangelos C. Fradelos, Ioanna V. Papathanasiou, Theodosios Paralikas, Ioanna Prasini, Maria Rekleiti, Aggeliki Katsapi, Maria Chatzi, Petros Galanis

**Affiliations:** 1 Department of Nursing, University of Thessaly, 41500 Larissa, Greece; 2 Clinical Epidemiology Laboratory, Faculty of Nursing, National and Kapodistrian University of Athens, 11527 Athens, Greece; 3 Home Care Nursing Department, Palliative Care Galilee, 19004 Spata, Greece; 4 Emergency Department, Andreas Syggros Hospital of Cutaneous and Venereal Diseases, 16121 Athens, Greece; 5 Euro-Mediterranean Institute for Quality and Safety in Health Services, 10678 Athens, Greece

**Keywords:** work environment, job burnout, turnover intention, nurses, Greece

## Abstract

**Background:**

A nurse's work environment has consistently been the most important factor affecting the emergence of burnout and their choice to resign from their positions.

**Objective:**

This work seeks to investigate the impact of the nursing work environment on job burnout and turnover intentions among nurses.

**Methods:**

A cross-sectional study was conducted during October 2024 in Greece. We employed the “Practice Environment Scale-5” to assess the nursing work environment. Additionally, we used the single item burnout measure to measure job burnout, and the single item turnover intention measure to measure turnover intention among our nurses. We used multivariable regression models to adjust for demographic and job variables.

**Results:**

More than half of the nurses (56.7%) reported a high level of turnover intention. The mean score on the single item burnout measure (7.78) indicated high levels of burnout in our sample. The multivariable linear regression analysis showed that lower levels of staffing and resource adequacy were associated with the increased job burnout (adjusted beta = −0.431, 95% *CI* = −0.683 to −0.180, *p*-value = 0.001). Similarly, our multivariable logistic regression model found an independent negative effect of staffing and resource adequacy on the turnover intention (adjusted *OR* = 0.594, 95% confidence interval = 0.421 to 0.840, *p*-value = 0.003).

**Conclusion:**

Our multivariable analysis indicated that a diminishing nursing work environment is associated with an increased job burnout and turnover intention. Improving the nursing work environment is essential to reduce the job burnout and turnover intention among nurses.

## 1. Introduction

The setting in which nurses deliver their services influences their occupational wellbeing and their choice to leave from their position. Researchers first focused on the working environment of nurses several decades ago, as a significant number of nurses were leaving their positions in hospitals, whereas certain hospitals, known as Magnet hospitals, experienced low resignation rates and successfully recruited new nursing personnel [1]. Interviews with nursing personnel and those in administrative positions documented the specific characteristics that differentiated Magnet hospitals, thereby encompassing administration, professional practice, and professional development. Specifically, these attributes encompassed a leadership approach that engages with and endorses personnel, and has sufficient staffing levels, advancement prospects, autonomy, mentorship, professional acknowledgment, respect, opportunities for professional growth, and formal education [2].

Despite nurses acknowledging the significance of the aforementioned characteristics, numerous healthcare organizations have yet to establish a positive working environment for their nursing personnel. Abusive leadership is prevalent in numerous nursing departments, which adversely impacts a nurse's performance, health, well-being, and the quality of nursing care [3]. Simultaneously, engaged leadership that strengthens, connects, empowers, and inspires positively impacts a nurse's work engagement, innovative behavior, and reduces their quiet quitting [4],[5]. When the organization and the nursing department supervisor acknowledge a nurse's contributions, establish conducive working conditions, and support staff in navigating challenging circumstances, the probability of nurses encountering burnout and indicating a desire to resign diminishes [6],[7].

Adequate nursing staffing has historically been one of the most critical resources that health care organizations have struggled to obtain. Prior to the onset of the COVID-19 pandemic, numerous healthcare organizations exhibited understaffed nursing departments, where nurses faced significant burnout, with understaffing identified as a contributing factor for their turnover intention [8]–[10]. Additionally, insufficient staffing adversely impacts the quality and safety of healthcare delivery [11]. Facilitating the ongoing professional development of nurses is essential to maintain the quality and patient safety. This can be accomplished in a conducive work environment marked by sufficient staffing, managerial support, robust enabling leadership, and a favorable workplace culture [12],[13].

Among the challenges of occupational well-being faced by nurses, burnout continuously exhibits the greatest prevalence rates, with one in three nurses indicating its occurrence [14]. Nurses are the professional group most impacted by burnout, as they experience the greatest rates of burnout among healthcare professionals [15]. Insufficient staffing in nursing departments, a lack of material resources, poor interpersonal interactions, and inadequate support from nursing leadership are key organizational variables that contribute to nurse burnout [16]–[19]. Additional organizational outcomes linked to nurse burnout encompass diminished work commitment, productivity, turnover intention, and self-efficacy [20]–[22]. The exhaustion of nurses jeopardizes the safety of healthcare services, as patients in departments staffed by exhausted nurses are more prone to various adverse outcomes, including bleeding, medication errors, infections, patient falls, and hypotension [20],[23].

In addition to the demanding working conditions caused by understaffing in nursing departments, nurses are leaving their jobs or resigning from the profession at high rates, which disrupts the efficient operation of nursing departments and places a financial burden on healthcare organizations [24],[25]. The global nurse turnover rate fluctuates between 8% and 36.6%, with significant variations across countries and diverse workplaces, often resulting in considerably at high rates, reaching up to 50% [26],[27]. The attributes of the work environment can negatively influence nurses' turnover intention, with burnout acting as a mediator variable between the work environment and turnover intention [28],[29].

The evaluation of a nurse's work environment encompasses critical elements that can influence their occupational well-being, and it is currently acknowledged as a significant instrument, as supported by numerous research, including studies conducted in Greece [18],[28]–[30]. In this context, we performed a study to investigate the effect of a nurse's work environment on job burnout and turnover intention in a sample of nurses in Greece.

## 2. Materials and methods

### 2.1. Study design

A cross-sectional study was conducted in Greece. Data gathering was conducted during October 2024. We utilized a web-based methodology to gather our data, thereby developing an online version of the study questionnaire using Google Forms, which we then disseminated to nursing communities on Facebook, Instagram, and LinkedIn. Consequently, our sample constituted a convenience sample. Nurses with a minimum of one year of clinical experience were eligible to participate in our study.

The sample size was determined using G*Power, version 3.1.9.2. We applied the following parameters in the calculation: (a) confidence level equal to 95%; (b) margin of error equal to 1%; (c) number of independent variables equal to 10 (five predictors and five confounders); and (d) low effect size (*f^2^* = 0.05) of nurse work environment on job burnout and turnover intention. We calculated a sample size of 370 nurses.

### 2.2. Instruments

We assessed the subsequent demographic and occupational variables: gender (female or male), age (continuous variable), employment in an inadequately staffed ward (yes or no), work in shifts (yes or no), and job experience (continuous variable).

The nurse's work environment was assessed using the “Practice Environment Scale-5.” (PES-5) [31]. The PES-5 is comprised of five components that assess five characteristics of the work environment: (a) nurse participation in hospital affairs; (b) nurse manager ability, leadership, and support; (c) collegial nurse-physician relationships; (d) staffing and resource adequacy; and (e) nursing foundations for quality of care. The answers are provided on a four-point Likert scale: completely disagree (1), disagree (2), agree (3), and completely agree (4). Higher values in the five dimensions are indicators of a better nursing work environment. The valid Greek version of the PES-5 was used [32]. We found that Cronbach's alpha for the PES-5 was 0.611.

We measured job burnout with the single item burnout measure [33]: “On a scale from 0 (not at all) to 10 (totally), how tired do you feel because of your job?”. Elevated values represent an increased degree of job burnout. The single item burnout was translated and validated in the Greek language [34].

We used the single item turnover intention measure to measure levels of turnover intention among our nurses [35]: “How often have you seriously considered leaving your current job?”. The participants can answer on a scale from 1 (rarely) to 6 (extremely often). The participants with a score ≥4 belong to the group with a high level of turnover intention, while the participants with a score <4 belong to the group with a low level of turnover intention.

### 2.3. Ethics

We implemented the principles of the Declaration of Helsinki to perform our study [36]. Furthermore, our study protocol received approval from the Ethics Committee of the Faculty of Nursing, National and Kapodistrian University of Athens (approval number; 01, September 26, 2024). Additionally, we provided the nurses with an information sheet which detailed the study design, and subsequently inquired if they wished to complete the study questionnaire.

### 2.4. Statistical analysis

We present categorical variables with numbers (percentages), and we use mean, standard deviation (*SD*), median, range, and interquartile range to present continuous variables. The distribution of continuous variables was assessed with the Kolmogorov-Smirnov test and Q-Q plots. We considered the five dimensions of the PES-5 as the independent variables. Additionally, we considered scores on job burnout and turnover intention measures as the dependent variables. PES-5 scores followed normal distribution, and, thus, we used a linear regression analysis to eliminate confounders. As we mentioned above, the score on the turnover intention measurement separates the participants into two groups: those with a low level of turnover intention and those with a high level of turnover intention. For this case, we used a logistic regression analysis to identify predictors of turnover intention. First, we performed either a univariate linear or a logistic regression analysis, and then we constructed a final multivariable linear or logistic regression model including all independent variables. Multivariable models were adjusted for the demographic and job variables. For the logistic regression analysis, we present unadjusted and adjusted odds ratios (*OR*), 95% *CI*, and *p*-values. For the linear regression analysis, we present unadjusted and adjusted coefficients beta, 95% confidence intervals (*CI*), and *p*-values; additionally, we used variance inflation factors (VIFs) to assess multicollinearity in the multivariable models. A VIF greater than 5 indicates multicollinearity between independent variables. Additionally, we produced a histogram of the regression standardized residuals to check for normality and calculated *R^2^* for the final multivariable linear regression model. For the final multivariable logistic regression model, we calculated Tjur's D that takes values from 0 to 1. *P*-values less than 0.05 were considered as statistically significant. We used the IBM SPSS, 21.0 (IBM Corp. Released 2012. IBM SPSS Statistics for Windows, Version 21.0. Armonk, NY: IBM Corp.) for the statistical analyses.

## 3. Results

### 3.1. Demographics

The sample was comprised of 388 nurses. The majority of nurses were females (88.4%, *n* = 343). The mean age of our sample was 40.9 years. The majority of nurses indicated that they practice in understaffed units (82.0%) and shifts (72.7%). The mean years of work experience was 16.0 years. Detailed demographic characteristics are shown in Table 1.

**Table 1.** Demographics of the study sample (*n* = 388).

**Demographics**	***N***	**%**
Gender		
Females	343	88.4
Males	45	11.6
Age	40.9 (10.0)^a^	41.0 (38.0)^b^
Understaffed units		
No	70	18.0
Yes	318	82.0
Shift work		
No	106	27.3
Yes	282	72.7
Work experience	16.0 (10.2)^a^	15.0 (17.0)^b^

Note: ^a^ mean, standard deviation; ^b^ median (range).

### 3.2. Study scales

In Table 2, we present descriptive statistics for the study scales. Regarding the nurse's work environment, dimensions of “collegial nurse-physician relationships”, “nurse manager ability, leadership, and support”, and “nursing foundations for quality of care” indicated a better work environment than dimensions of “staffing and resource adequacy” and “nurse participation in hospital affairs”.

The mean score on the single item burnout measure indicated high levels of burnout. When applying the suggested cut-off point for the single item turnover intention measure, more than half of the nurses (56.7%, *n* = 220) reported a high level of turnover intention, while 43.3% (n=168) reported a low level of turnover intention.

**Table 2.** Descriptive statistics for the study scales.

**Scales factors**	**Mean**	**Standard deviation**	**Median**	**Range**	**Interquartile range**
Practice Environment Scale-5					
Nurse participation in hospital affairs	1.65	0.68	2.00	3.00	1.00
Nursing foundations for quality of care	2.15	0.77	2.00	3.00	1.00
Staffing and resource adequacy	1.72	0.77	2.00	3.00	1.00
Collegial nurse-physician relationships	2.49	0.72	3.00	3.00	1.00
Nurse manager ability, leadership, and support	2.28	0.87	2.00	3.00	1.00
Single item burnout measure	7.78	1.74	8.00	10.00	2.00
Single item turnover intention measure	3.91	1.62	4.00	5.00	2.00

### 3.3. Impact of nurse work environment on job burnout

**Figure 1.**
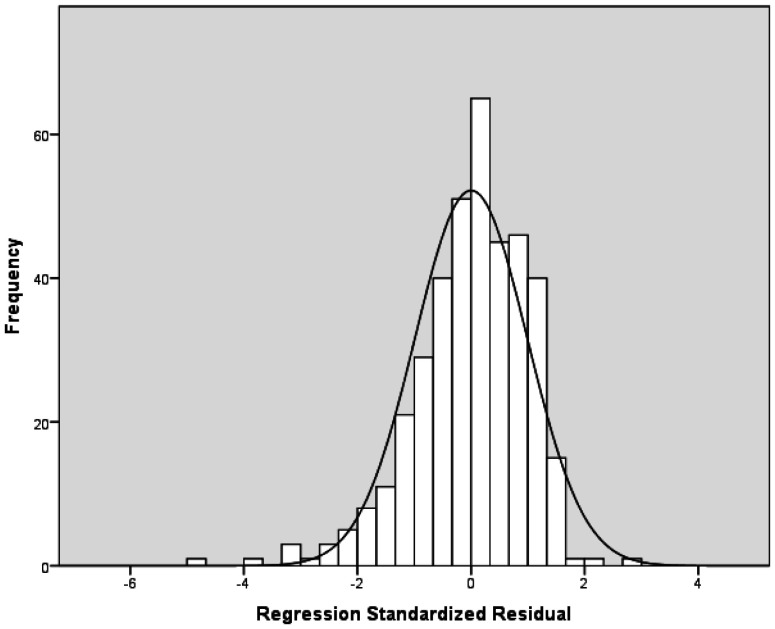
Histogram of the residuals with job burnout as the dependent variable.

Table 3 presents the linear regression analysis with job burnout as the dependent variable. The univariate linear regression analysis showed that the nurse's participation in hospital affairs, staffing and resource adequacy, and collegial nurse-physician relationships were associated with job burnout. However, the multivariable linear regression analysis showed that only staffing and resource adequacy had an independent effect on job burnout. In particular, lower levels of staffing and resource adequacy were associated with an increased occurrence of job burnout. The VIFs for the independent variables ranged from 1.008 to 1.543, thus indicating no multicollinearity between the independent variables. A histogram of the regression standardized residuals denoted normal distribution of residuals is presented in Figure 1.

**Table 3.** Linear regression analysis with job burnout as the dependent variable.

**Independent variables**	**Univariate model**		**Multivariable model^a^**	
	**Unadjusted coefficient beta (95% *CI*)**	***P*-value**	**Adjusted coefficient beta (95% *CI*)**	***P*-value**
Nurse participation in hospital affairs	−0.323 (−0.578 to −0.067)	0.013	−0.027 (−0.282 to 0.228)	0.834
Nursing foundations for quality of care	−0.195 (−0.420 to 0.031)	0.090	−0.026 (−0.262 to 0.211)	0.832
Staffing and resource adequacy	−0.730 (−0.943 to −0.516)	<0.001	−0.431 (−0.683 to −0.180)	0.001
Collegial nurse-physician relationships	−0.376 (−0.616 to −0.137)	0.002	−0.192 (−0.432 to 0.048)	0.117
Nurse manager ability, leadership, and support	−0.187 (−0.387 to 0.014)	0.068	−0.108 (−0.312 to 0.097)	0.302

Note: *CI*: confidence interval; ^a^ Multivariable linear regression model is adjusted for gender, age, understaffed ward, shift work, and work experience. R^2^ for the multivariable model was 19.6%.

### 3.4. Impact of nurse work environment on turnover intention

Table 4 shows results from the logistic regression analysis with turnover intention as the dependent variable. The univariate logistic regression analysis identified a statistically significant association between all dimensions of the nurse's work environment and turnover intention. However, after adjustment for confounders, we found that only staffing and resource adequacy were associated with turnover intention. In particular, lower levels of staffing and resource adequacy were associated with a higher probability of turnover intention. Tjur's R^2^ for the multivariable model was 0.29.

**Table 4.** Logistic regression analysis with turnover intention as the dependent variable (reference category = low level of turnover intention).

**Independent variables**	**Univariate model**		**Multivariable model^a^**	
	**Unadjusted *OR* (95% *CI*)**	*P*-value	Adjusted *OR* (95% *CI*)	*P*-value
Nurse participation in hospital affairs	0.623 (0.459 to 0.845)	0.002	0.856 (0.606 to 1.208)	0.375
Nursing foundations for quality of care	0.584 (0.444 to 0.768)	<0.001	0.738 (0.531 to 1.024)	0.069
Staffing and resource adequacy	0.532 (0.402 to 0.703)	<0.001	0.594 (0.421 to 0.840)	0.003
Collegial nurse-physician relationships	0.649 (0.486 to 0.868)	0.004	0.797 (0.570 to 1.113)	0.183
Nurse manager ability, leadership, and support	0.716 (0.564 to 0.908)	0.006	0.881 (0.666 to 1.165)	0.374

Note: *CI*: confidence interval; *OR*: odds ratio; ^a^ Multivariable logistic regression model is adjusted for gender, age, understaffed ward, shift work, and work experience. Tjur's R^2^ for the multivariable model was 0.29.

## 4. Discussion

This study assessed the occupational environment of nurses, their degree of burnout, and their turnover intention. The findings emphasized that collegial nurse-physician relationships and the abilities, leadership, and support of nurse managers were the most favorable attributes, whereas staffing and resource adequacy earned the lowest evaluations. These results align with those of other research [23],[30],[37]. Physicians and nurses are frontline healthcare professionals and provide nearly all of the care that patients receive. Effective communication, cooperation, and teamwork between nurses and physicians are crucial to a nurse's occupational well-being, their willingness to remain in the profession, their performance, and the quality and safety of the health services provided [38]–[40]. Alongside the collaborative interactions between nurses and physicians, essential aspects of the nursing work environment, including nursing leadership and the accessibility of both human and material resources, can influence the outcomes for nurses, patients, and the organization [41]. Nursing leaders who empower their personnel, enhance engagement, improve the workplace the environment, and apply leadership styles such as transformational or authentic leadership diminish the probability of the staff experiencing burnout [42]. The transformational leadership style and strong working relationships between physicians and nurses are predictor factors for the nurse's low turnover intention [43],[44]. A recent study indicated that poor collegial relationships between nurses and physicians, together with limited leadership and support from nursing supervisors, were evident. These conditions were linked to higher occurrences of quiet quitting among nurses and lower levels of work engagement. [45]. Although nurses opt for quiet quitting as a form of self-protection against their demanding work environment, which they perceive as unimproved, this choice does not prevent them from leaving their jobs at the first opportunity [46]. Furthermore, as work engagement decreases, the nurse's turnover intention increases [47].

The study revealed that participants exhibited significant burnout levels and elevated turnover intention rates. Furthermore, the attributes of their work environment, including reduced personnel levels and insufficient resources, were associated with a heightened likelihood of turnover intention and elevated job burnout. Similar findings were reported in a meta-analysis regarding the nurse-to-patient ratio [48]. Nurses suggest improving staffing levels as a very important intervention to reduce their burnout and improve their well-being [49]. Furthermore, nurses recognize the workload due to understaffing as an important factor that pushes them to quit their jobs [50]. Although nurses acknowledge staffing as a vital component of their work environment, organizational management does not seem to regard this problem with comparable significance. A considerable body of recent research has highlighted understaffing as a determinant that influences a nurse's workplace well-being and their propensity to resign [27],[51],[52]. Nursing understaffing leads to burnout and turnover intention, while burnout further increases the intention to leave and turnover worsens staffing levels. This creates a vicious cycle that traps nurses in an unhealthy work environment where organizational pathologies are perpetuated. Moreover, a significant challenge for healthcare systems is the rising prevalence of chronic diseases, which may further exacerbate the nursing workload in the context of understaffing. Patients now present with more complex care needs that extend beyond a single underlying condition and often requires the management of two or even three chronic illnesses or conditions [53],[54]. The combination of inadequate staffing and increasing care demands may contribute to heightened levels of job burnout and an increased turnover intention among nurses.

## 5. Limitations

There is a need to mention the limitations of our study. Initially, we employed a cross-sectional design for our study; hence, we were unable to ascertain causal correlations among the nursing work environment, job burnout, and turnover intention. Second, we employed a convenience sample through social media platforms; therefore, we could not extrapolate our findings to the broader population of Greek nurses. For instance, we had a low percentage of males in our study. Third, we used self-reported tools to measure the nurse's perceptions of the nursing work environment, job burnout, and turnover intention. In this context, information bias is probable in our study. Objective indicators of work-related variables, such as nurse staffing levels, may resolve this issue in future studies. Fourth, we eliminated several confounders in our multivariable regression analysis. However, several other variables (e.g., unit type, case mix, nursing care complexity, financial stress, organizational policy, personal health) may act as confounders in the relationship between the nursing work environment, job burnout, and turnover intention and should be adjusted in future studies. An important parameter of the present study, which may have influenced our outcomes, is the fact that the participant recruitment was conducted through social media platforms. Evidence suggests that employees, and particularly nurses, who actively use social networking platforms report lower levels of burnout and turnover intention [55]–[57]. It is likely that communication, interaction, counseling, and mutual support among nurses within these networks play a crucial supportive role, thereby mitigating occupational burnout while simultaneously reducing their intention to leave the profession. Therefore, our findings might have been different (potentially indicating even higher levels of burnout and turnover intention) if the participant recruitment was performed through face-to-face contact. Apart from understaffing, another factor that increases a nurse's workload is the severity and complexity of the hospitalized patients [58],[59]. Therefore, staffing levels and the nurse-to-patient ratio alone do not fully reflect a nurse's workload, and the complexity of the patient cases should also be taken into account when assessing workload.

Additionally, we used single-items measures to assess job burnout and turnover intention. In recent years, the development of single-item measurement scales, as well as the publication of short versions of previously established multi-item scales, has been gaining increasing attention. Although single-item scales have been criticized with respect to their reliability and their potential inability to capture multidimensional constructs, these concerns have not been conclusively addressed by a substantial body of research [60]–[63]. Moreover, single-items use may increase the response rate since the participants need less time to fill in the questionnaire. This is another reason that we chose to use single-item measures. Although these tools are valid, they cannot capture the multidimensional nature of job burnout and turnover intention. Therefore, future studies should use multi-item scales to measure these variables (e.g., Maslach Burnout Inventory, Copenhagen Burnout Inventory, multi-item turnover measures) and complement subjective measures with objective workload indicators (e.g., patient acuity, staffing ratios).

Finally, Cronbach's alpha for the PES-5 was below the recommended threshold. Although the PES-5 is a well-established tool to measure the nursing work environment, further studies should be conducted in the Greek context to examine the reliability and validate of the scale with more representative and random samples. Therefore, additional studies with larger samples are required to further validate the cultural adaptation and contextual validation of the tool.

## 6. Conclusions

The work environment in which nurses operate predicts their occupational well-being and their purpose to resign from their positions. This study emphasized that staffing and accessible resources are the most significant factors in the work environment that affect the burnout levels and the intention to resign. Inadequate staffing is a significant and persistent challenge that healthcare organizations appear to struggle to resolve. The well-being of nurses is crucial for the quality of care provided and their likelihood to resign from their roles. Thus, fostering a healthy work environment provides multiple benefits for patients, nurses, and organizational efficiency, thus rendering its improvement a managerial priority.

## Use of AI tools declaration

The authors declare they have not used Artificial Intelligence (AI) tools in the creation of this article.

## References

[1] McClure ML, Poulin MA, Sovie MD (1983). Magnet hospitals: Attraction and retention of professional nurses, Kansas City, Missouri, American Academy of Nursing.

[2] Magnet Hospitals (1983). Attraction and retention of professional nurses. Task Force on Nursing Practice in Hospitals. American Academy of Nursing. ANA Publ.

[3] Labrague LJ (2024). Abusive supervision and its relationship with nursing workforce and patient safety outcomes: A systematic review. West J Nurs Res.

[4] Moisoglou I, Katsiroumpa A, Papathanasiou IV (2025). Engaging leadership reduces quiet quitting and improves work engagement: Evidence from nurses in Greece. Nurs Rep.

[5] Moisoglou I, Katsiroumpa A, Prasini I (2024). Innovation support reduces quiet quitting and improves innovative behavior and innovation outputs among nurses in Greece. Nurs Rep.

[6] Chami-Malaeb R (2021). Relationship of perceived supervisor support, self-efficacy and turnover intention, the mediating role of burnout. Pers Rev.

[7] Galanis P, Moisoglou I, Papathanasiou IV (2024). Association between organizational support and turnover intention in nurses: A systematic review and meta-analysis. Healthcare.

[8] Bae SH (2024). Assessing the impacts of nurse staffing and work schedules on nurse turnover: A systematic review. Int Nurs Rev.

[9] Lasater KB, Aiken LH, Sloane DM (2021). Chronic hospital nurse understaffing meets COVID-19: An observational study. BMJ Qual Saf.

[10] Galanis P, Vraka I, Fragkou D (2021). Nurses' burnout and associated risk factors during the COVID-19 pandemic: A systematic review and meta-analysis. J Adv Nurs.

[11] Kiekkas P, Tsekoura V, Aretha D (2019). Nurse understaffing is associated with adverse events in postanaesthesia care unit patients. J Clin Nurs.

[12] Coventry TH, Maslin-Prothero SE, Smith G (2015). Organizational impact of nurse supply and workload on nurses continuing professional development opportunities: An integrative review. J Adv Nurs.

[13] King R, Taylor B, Talpur A (2021). Factors that optimise the impact of continuing professional development in nursing: A rapid evidence review. Nurs Edu Today.

[14] Ge MW, Hu FH, Jia YJ (2023). Global prevalence of nursing burnout syndrome and temporal trends for the last 10 years: A meta-analysis of 94 studies covering over 30 countries. J Clin Nurs.

[15] Galanis P, Moisoglou I, Katsiroumpa A (2023). Increased job burnout and reduced job satisfaction for nurses compared to other healthcare workers after the COVID-19 pandemic. Nurs Rep.

[16] Bae SH (2021). Intensive care nurse staffing and nurse outcomes: A systematic review. Nursing Crit Care.

[17] Sillero A, Zabalegui A (2018). Organizational factors and burnout of perioperative nurses. Clin Pract Epidemiol Ment Health.

[18] Moisoglou I, Yfantis A, Tsiouma E (2021). The work environment of haemodialysis nurses and its mediating role in burnout. J Renal Care.

[19] Muir KJ, Porat-Dahlerbruch J, Nikpour J (2024). Top factors in nurses ending health care employment between 2018 and 2021. JAMA Netw Open.

[20] Dall'Ora C, Ball J, Reinius M (2020). Burnout in nursing: A theoretical review. Hum Resour Health.

[21] Chang HY, Friesner D, Chu TL (2018). The impact of burnout on self-efficacy, outcome expectations, career interest and nurse turnover. J Adv Nurs.

[22] Jun J, Ojemeni MM, Kalamani R (2021). Relationship between nurse burnout, patient and organizational outcomes: Systematic review. Int J Nurs Stud.

[23] Prezerakos P, Galanis P, Moisoglou I (2015). The work environment of haemodialysis nurses and its impact on patients' outcomes. Int J Nurs Pract.

[24] Sawada S, Takemura Y, Isobe T (2022). Perceived impact of nurse turnover on the organization: A Delphi study on managers of nursing. J Nurs Manag.

[25] Bae SH (2022). Noneconomic and economic impacts of nurse turnover in hospitals: A systematic review. Int Nurs Rev.

[26] Woodward KF, Willgerodt M (2022). A systematic review of registered nurse turnover and retention in the United States. Nurs Outlook.

[27] Galanis P, Moisoglou I, Katsiroumpa A (2025). Workload increases nurses' quiet quitting, turnover intention, and job burnout: Evidence from Greece. AIMS Public Health.

[28] Poku CA, Donkor E, Naab F (2022). Impacts of nursing work environment on turnover intentions: The mediating role of burnout in Ghana. Nurs Res Prac.

[29] Wan Q, Li Z, Zhou W (2018). Effects of work environment and job characteristics on the turnover intention of experienced nurses: The mediating role of work engagement. J Adv Nur.

[30] Dutra CK dos R, Guirardello E de B (2021). Nurse work environment and its impact on reasons for missed care, safety climate, and job satisfaction: A cross-sectional study. J Adv Nurs.

[31] Lake ET, Gil J, Moronski L (2024). Validation of a short form of the practice environment scale of the nursing work index: The PES-5. Res Nurs Health.

[32] Katsiroumpa A, Moisoglou I, Konstantakopoulou O (2024). Practice environment scale of the nursing work index (5 items version): Translation and validation in Greek. Int J Caring Sci.

[33] Hansen V, Pit S (2016). The single item burnout measure is a psychometrically sound screening tool for occupational burnout. Health Scope.

[34] Galanis P, Katsiroumpa A, Vraka I (2024). The single item burnout measure is a reliable and valid tool to measure occupational burnout. Arch Hell Med.

[35] Spector PE, Dwyer DJ, Jex SM (1988). Relation of job stressors to affective, health, and performance outcomes: A comparison of multiple data sources. J Appl Psychol.

[36] World Medical Association (2013). World medical association declaration of Helsinki: Ethical principles for medical research involving human subjects. JAMA.

[37] Moisoglou Ioannis, Yfantis Aris, Galanis Petros (2020). Nurses work environment and patients' quality of care. Int J Caring Sci.

[38] Labrague LJ (2025). A systematic review on nurse-physician collaboration and its relationship with nursing workforce outcomes: Implications for nursing administration. J Nurs Adm.

[39] Kang XL, Brom HM, Lasater KB (2020). The association of nurse–physician teamwork and mortality in surgical patients. West J Nurs Res.

[40] Boev C, Xia Y (2015). Nurse-physician collaboration and hospital-acquired infections in critical care. Crit Care Nurse.

[41] Cristina Gasparino R, Daiana Mendonça Ferreira T, Ceretta Oliveira H (2021). Leadership, adequate staffing and material resources, and collegial nurse–physician relationships promote better patients, professionals and institutions outcomes. J Adv Nurs.

[42] Wei H, King A, Jiang Y (2020). The impact of nurse leadership styles on nurse burnout: A systematic literature review. Nurse Lead.

[43] Suliman M, Aljezawi M, Almansi S (2020). Effect of nurse managers' leadership styles on predicted nurse turnover. Nurs Manag (Harrow).

[44] Galletta M, Portoghese I, Battistelli A (2013). The roles of unit leadership and nurse–physician collaboration on nursing turnover intention. J Adv Nurs.

[45] Moisoglou I, Katsiroumpa A, Katsapi A (2025). Poor nurses' work environment increases quiet quitting and reduces work engagement: A cross-sectional study in Greece. Nurs Rep.

[46] Galanis P, Moisoglou I, Malliarou M (2024). Quiet quitting among nurses increases their turnover intention: Evidence from Greece in the post-COVID-19 era. Healthcare.

[47] Zhu LL, Wang HJ, Xu YF (2023). The effect of work engagement and perceived organizational support on turnover intention among nurses: A meta-analysis based on the price–Mueller model. J Nurs Manage.

[48] Shin S, Park JH, Bae SH (2018). Nurse staffing and nurse outcomes: A systematic review and meta-analysis. Nurs Outlook.

[49] Aiken LH, Lasater KB, Sloane DM (2023). Physician and nurse well-being and preferred interventions to address burnout in hospital practice: Factors associated with turnover, outcomes, and patient safety. JAMA Health Forum.

[50] Friese CR, Medvec BR, Marriott DJ (2024). Changes in registered nurse employment plans and workplace assessments. JAMA Netw Open.

[51] Haywood D, Crocker KM, Gnatt I (2024). What accounts for turnover intention in the Australian public mental health workforce?. Int J Ment Health Nurs.

[52] Jane Muir K, Sliwinski K, Pogue CA (2025). Lower burnout among hospital nurses in California attributed to better nurse staffing ratios. Policy Polit Nurs Pract.

[53] Huang SL, Cheng H, Duffield C (2021). The relationship between patient obesity and nursing workload: An integrative review. J Clin Nurs.

[54] Cesare M, D'Agostino F, Damiani G (2025). Exploring the impact of medical complexity on nursing complexity of care in paediatric patients: A retrospective observational study. J Clin Nurs.

[55] Zhang X, Ma L, Xu B (2019). How social media usage affects employees' job satisfaction and turnover intention: An empirical study in China. Inf Manag.

[56] Mao Y, Fu H, Feng Z (2020). Could the connectedness of primary health care workers involved in social networks affect their job burnout? A cross-sectional study in six counties, Central China. BMC Health Serv Res.

[57] Gu J, Zhu P, Chu Y (2025). Association between social media use and burnout among primary health care workers during the COVID-19 pandemic in China: Nationwide cross-sectional survey. J Med Internet Res.

[58] Ivziku D, Ferramosca FMP, Filomeno L (2022). Defining nursing workload predictors: A pilot study. J Nurs Manag.

[59] Cesare M, D'Agostino F, Sebastiani E (2025). Deciphering the link between diagnosis-related group weight and nursing care complexity in hospitalized children: An observational study. Children (Basel).

[60] Allen MS, Iliescu D, Greiff S (2022). Single item measures in psychological science. Eur J Psychol Assess.

[61] Castro MS, Bahli B, Ferreira JJ (2023). Comparing single-item and multi-item trust scales: Insights for assessing trust in project leaders. Behav Sci (Basel).

[62] Franke GR, Rapp A, “Mick” Andzulis J (2013). Using shortened scales in sales research: Risks, benefits, and strategies. J Pers Sell Sales M.

[63] Bergkvist L (2015). Appropriate use of single-item measures is here to stay. Mark Lett.

